# Plasma Isoagglutinin Depletion for Blood Group Independent Plasma Transfusion

**DOI:** 10.1159/000521217

**Published:** 2022-02-04

**Authors:** Johannes Raster, Michelle Jacob, Andreas Greinacher, Konstanze Aurich

**Affiliations:** Institut für Immunologie und Transfusionsmedizin, Abteilung Transfusionsmedizin, Universitätsmedizin Greifswald, Greifswald, Germany

**Keywords:** Universal plasma, Transfusion, Isoagglutinin depletion

## Abstract

**Background:**

Plasma transfusion is one of the basic treatments in patients with major blood loss. The anti-A and anti-B antibodies contained in the plasma demand ABO blood group compatibility. This is limiting the use of plasma in emergency situations and can cause a shortage in the supply of plasma of certain blood groups. We developed a method for anti-A and anti-B depletion by adsorbing plasma isoagglutinins using red blood cells.

**Materials and Methods:**

Three units of fresh frozen plasma were thawed after quarantine storage, pooled, and an aliquot of red cell concentrate was added. After 2 h of incubation at room temperature antibody-red-cell complexes were removed by centrifugation, the isoagglutinin-depleted plasma was split into three units and deep frozen. Isoagglutinin titers, free hemoglobin, residual red cells, clotting factor activity, and sterility of plasma units were determined after isoagglutinin depletion and a double freeze-thawing procedure.

**Results:**

Anti-B titers in group A plasma were reduced from values of 1:64 to 1:1 or lower, anti-A titers in group B plasma decreased from values of 1:128 to at least 1:16. Postprocedure clotting factor activities were preserved with 88.0 ± 7.3% (factor V), 106.9 ± 11.4% (factor VIII), and 84.0 ± 7.5% (factor XI) fulfilling the quality control requirements. No residual red cells were found, but free hemoglobin slightly increased to 53.7 ± 5.2 μmol/L. All units were sterile.

**Discussion:**

We described a method for the production of anti-A- and anti-B-depleted plasma in a closed system that uses standard equipment. The resulting isoagglutinin-depleted plasma may allow for blood group independent plasma transfusion.

## Introduction

Plasma is transfused to patients to treat and prevent coagulopathies, to replace labile coagulation factors during massive transfusion, liver diseases, or is a treatment for patients with thrombotic microangiopathies often as plasma exchange therapy [1, 2]. Transfused plasma needs to be ABO blood group compatible because anti-A and anti-B IgM and IgG antibodies (isoagglutinins) can cause adverse effects in recipients expressing the cognate antigens on endothelium and red cells [3]. The requirement of blood group compatibility is a limitation to treat bleeding emergencies in patients with an unknown ABO blood group (e.g., prehospital transfusions) [4]. Only group AB plasma, which does not contain isoagglutinins, can be transfused universally. However, as the prevalence for blood group AB in Germany is only about 6%, the access to AB plasma is limited despite targeted recruitment of AB plasma donors [5]. The need for AB plasma is consistently high as AB plasma is used as universal plasma and only 27% of AB plasma units transfused in 2014 in hospitals around the world were transfused to group AB recipients [6]. Three clinical situations are attractive for blood group independent transfusion of plasma. First are emergency situations transfusing the unknown patient, second is the use of convalescent plasma for newly emerging pathogens, and third is the much easier logistics for smaller hospitals, which would need to store only one type of plasma for all patients. Current emergency release protocols usually allow the use of group A plasma for patients of unknown blood group to overcome the shortage in AB plasma stocks [7]. Several emergency release protocols categorize the group A plasma units in anti-B high titer (≥1:128), low titer (≤1:64 but ≥1:16), and very low titer (<1:16) with decreasing risk of transfusion-related adverse effects [8]. However, these protocols compromise between accepting potential adverse effects of incompatible plasma transfusions and the benefit of being able to supply sufficient amounts of plasma to the patient [9]. Using group A plasma instead of AB requires extensive testing of multiple plasma units to identify the very low titer units eligible for emergency release. As most methods for anti-A and anti-B determination come with inter- and intra-laboratory variations of up to 4 titration steps, extensive titer testing might lead to a false sense of security [10, 11, 12]. Another, presumably more reliable, approach to reduce the risk of incompatible transfusions associated with the emergency release of therapeutic plasma is the depletion of isoagglutinins from therapeutic plasma. Current approaches to produce “universal plasma” by antibody depletion require either larger pools of plasma with different ABO blood groups or industrial scale processes producing batches of hundreds of liters [13, 14]. Both mentioned procedures utilize A- and B-antigens on soluble plasma proteins to form immune complexes effectively blocking the antigen binding site of the isoagglutinins. Such anti-A- and anti-B-depleted plasma has been used in military settings and during cardiac surgery [15, 16]. It is difficult to establish these procedures in small individual blood banks as they require either specialized or large-scale equipment. The principle of adsorbing the isoagglutinins onto structures expressing the cognate antigens can also be applied by adding red cells from red cell concentrates [17]. This also allows for the removal of the formed immune complexes. In the present study we developed a procedure for the production of small batches of anti-A- and anti-B-depleted plasma by isoagglutinin red blood cell adsorption using resources available in every blood bank facility.

## Materials and Methods

### Blood Collection and Processing

Plasma and red cell concentrate (RCC) were collected by fractionation of whole blood donations from voluntary healthy donors (centrifugation at 4,000 *g* for 10 min at 21°C; Roto Silenta 630 S, Hettich GmbH, Germany; separation by MacoPress Smart, Maco Pharma, France) after letting the whole blood rest for 1–8 h at room temperature in compliance with the German guideline for hemotherapy [18]. Whole blood was collected into a quadruple blood bag system T/B 500 mL, citrate phosphatedextrose solution systems with phosphate-adenine-glucose-guanosin-saline-mannitol solution (PAGGS-M) as additive solution (Maco Pharma).

The plasma units were not leukocyte depleted. All plasma units were shock frozen (HOF GmbH, Germany) within 6 h after fractionation at −60°C for 1 h and stored below −30°C. After quarantine storage for 4 months, the plasma units were thawed at 37°C (Plasmatherm, Barkey GmbH, Germany).

The RCC was leukocyte depleted by in-line filtration (LCRD2 Leukoflex, Maco Pharma, France), split into 45-mL aliquots using pediatric blood bags (Maco Pharma, France) and stored at 2–6°C. The red cell count of RCC was determined by a Sysmex cell counter (Sysmex XP-300, Sysmex Deutschland GmbH, Germany). All process steps are performed under GMP conditions.

### Determination of the Required Red Blood Cell Amount for Anti-A and/or Anti-B Depletion in Plasma

Initially, we used small volume plasma pools of 4 mL containing equal amounts of plasma from three donors to determine the amount of RCC required for sufficient anti-A and/or anti-B depletion of plasma (*n* = 15 group A plasma, *n* = 8 group B plasma, *n* = 10 group O plasma). Group B red cells were added to group A plasma pools, group A_1_ red cells to group B plasma, and group AB red cells to group O plasma pools. After incubation in polystyrol tubes (2 h; room temperature; standing upright; no agitation) the red cells were removed by centrifugation (10 min, 2,500 *g*) and the supernatant was used for further analysis. The RCC volumes required for small volume plasma samples were extrapolated to one full plasma unit (300 mL). Samples were analyzed for isoagglutinin titer directly after treatment.

### Anti-A and Anti-B Titer Determination

Titers of anti-A and anti-B were determined by a microcolumn gel card system. For anti-A and anti-B IgM, saline cards (ID-Card for sodium chloride, enzyme test and cold agglutinins; Bio-Rad Laboratories Inc., USA) were used; for anti-A and anti-B IgG cards with anti-human globulin phase (ID-Card LISS/Coombs, Bio-Rad Laboratories Inc.) were used according to the manufacturer's instructions. In brief, plasma samples in 1:2 dilution series (dilution media: 0.9% NaCl solution) were incubated (room temperature [IgM] or 37°C [IgG], 15 min) with group B red cells for anti-A and group A red cells for anti-B. Group O red cells were used as a negative control. Agglutination strengths were evaluated by two independent individuals. Discrepant assessment results were solved by discussion. Antibody titer was defined as the last plasma dilution inducing agglutination.

### Other Quality Parameters

Residual red cells in anti-A- and anti-B-depleted plasma units were counted using a Nageotte chamber (Assistent, Sonderheim vor der Rhön, Germany) under a microscope. Clotting factor V, VIII, and XI activities were analyzed using 500 μL of supernatant from centrifuged plasma on a CS5100 device (Siemens Healthcare, Germany) by standard methods based on factor-deficient plasma.

Free hemoglobin, as a marker for hemolysis of red cells used for isoagglutinin adsorption, was measured as cyanmethemoglobin spectroscopically using potassium hexacyanoferrate III and potassium cyanide (= transformation reagent) in cell free supernatant. A multi-wavelength absorption analysis was performed at 540 nm (A1) and 680 nm (A2) in a spectrophotometer (UV-1700, Shimadzu, Japan).

Free hemoglobin content of untreated plasma units prepared for routine quality control served as the reference (*n* = 10). Microbiological control of the treated plasma units was assessed according to method 2.6.27 of the European Pharmacopoeia [19].

### Development of a Bag System for Anti-A/Anti-B Depletion in a Closed System

The bag system for the production of anti-B-depleted plasma from group A plasma units consisted of two 500-mL bags (Maco Pharma, France) connected to four blunt ends via several standard Y-connectors (Maco Pharma) which were connected using sterile docking (TSCDII, Terumo BCT, Belgium). All processing steps required were carried out in a closed system.

**Fig. 1:**
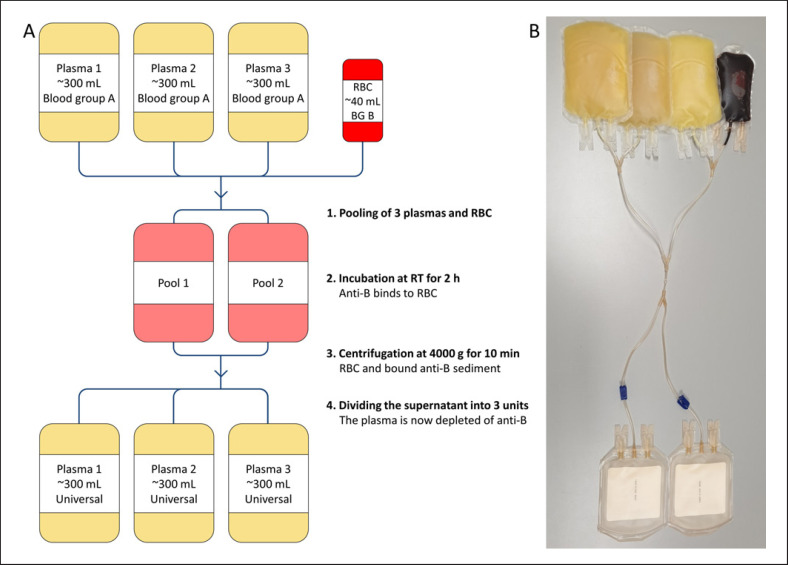
**A** Schematic of the process of the production of universal plasma. BG, blood group. **B** Device configuration used to produce anti-A- and anti-B-depleted plasma.

Three plasma units (280–330 mL each) were thawed (Plasmatherm, Barkey, Germany) and pooled in two 500-mL bags with simultaneous addition of the 45-mL RCC aliquot using sterile docking (TSCDII, Terumo, Brussels, Belgium). The plasma/RCC pool was incubated for 2 h at room temperature, centrifuged (4,000 *g*, 10 min, Hettich Roto Silentia 630 RS, Hettich GmbH, Germany) and separated by a manual press (Fenwal® Plasma Extractor, Baxter Healthcare Corporation, USA). The supernatant was transferred into the original plasma bags. Plasma units were frozen in a shock freezer (60 min, −60°C, 2 bar, HOF GmbH, Germany) and stored at −30 (+3)°C until use. A schematic of the process and the bag system are given in Figure 1. Samples were analyzed for isoagglutinin titer directly after treatment. Residual cell count, free hemoglobin, and clotting factor V, VIII, and XI activity were determined after storage at −30°C.

## Results

**Fig. 2:**
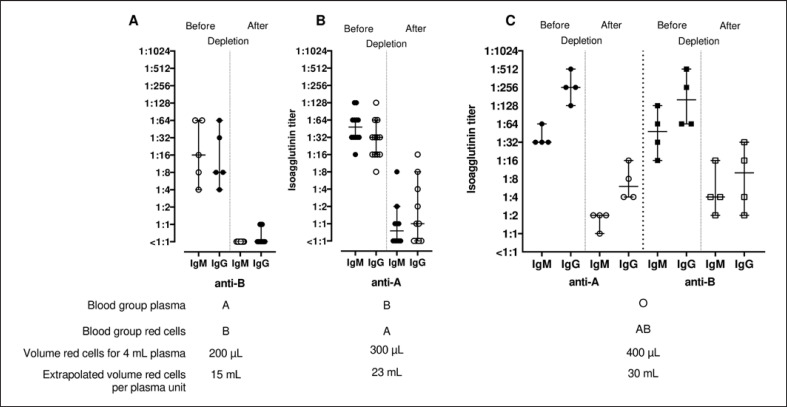
Isoagglutinin IgM and IgG titers before and after treating small batches (4 mL) of group A plasma with group B red cells (**A**), group B plasma with group A red cells (**B**), and group O plasma with group AB red cells (**C**). The volume of red cells per plasma unit is extrapolated from isoagglutinin depletion in 4 mL of plasma. Medians and 95% confidence intervals are given.

Before the production of isoagglutinin-depleted plasma units from quarantine stored fresh frozen plasma, we determined the required RCC volume by measuring the titers in a small volume approach of 4 mL of plasma and extrapolated the results for plasma units of 300 mL. A volume of 200 μL of group B RCC was found to be sufficient to reduce the IgG and IgM anti-B titers in 4 mL of the group A plasma pool to <1:2 (Fig. 2A). For plasma pools of group B, 300 μL of group A RCC was required to decrease the IgG and IgM anti-A titers to values of 1:16 or lower (Fig. 2B). A volume of 400 μL of group AB RCC was required to reduce the anti-A and anti-B titers to values of 1:16 or lower for group O plasma units with the exception of one pool, where anti-B IgG titer was not decreased below 1:32 (Fig. 2C). Extrapolating the RCC volumes to one whole plasma unit (300 mL), we required 15 mL of group B RCC to treat a unit of group A plasma, 23 mL of group A RCC to treat a unit of group B plasma, and at least 30 mL of group AB RCC to treat a unit of group O plasma.

The process was scaled up to a closed bag system and used to produce 4 pools (12 units) of anti-B-depleted plasma from group A plasma by pooling three plasma units and adsorbing anti-B onto red cells of group B RCC. We used the extrapolated RCC volume determined in the small volume batch and added this to the plasma pool. Anti-B IgM and IgG in all four group A plasma pools were reduced to titers of 1:1 or lower. No bacterial contamination was detected (Table 1). Most of the quality parameters were not affected by the isoagglutinin depletion and the double freeze-thawing process compared to non-treated plasma units (Table 2). The isoagglutinin-red cell aggregates were completely removed by centrifugation. Coagulation factor V, VIII, and XI activity levels were also maintained. Cell-free hemoglobin increased by twice the baseline value (Table 2) but remained within the accepted quality range [20].

## Discussion

We present a method to produce isoagglutinin-depleted plasma in a closed bag system. The method is highly efficient and even outliers did not exceed isoagglutinin titers of 1:16, which are below the risk of severe transfusion-related adverse effects as observed for non-processed plasma [21, 22] and former commercially available Bioplasma FDP and Uniplas®, where IgM and IgG titers of <1:32 did not result in hemolysis [23, 24]. Due to the large RCC volume required for the isoagglutinin depletion of group O plasma (30 mL for one plasma unit), we concentrated on group A plasma.

When comparing and interpreting data regarding safe titers of isoagglutinins obtained from different studies and guidelines [8, 9, 23, 24], variations regarding the methods have to be considered. We used the gel microcolumn hemagglutination technique, which is more sensitive and less variable than the commonly used tube technique [11]. Even taking the possible inaccuracies of the method into account, the remaining isoagglutinin titers were so low that the risk for isoagglutinin-mediated transfusion-related adverse effects seems to be negligible.

The quality of the isoagglutinin-depleted plasma remains high. Despite the double freezing and thawing process during manufacturing, clotting factor V, VIII, and XI activity was preserved and within the specification range of conventional fresh frozen plasma [25]. Although we depleted all red cells used for adsorption, as assessed by the highly sensitive nageotte chamber method, formation of red cell microvesicles and thus the risk of allo-senzitation against erythrocyte antigens cannot be excluded. This is highlighted by the observed red cell lysis indicated by the increase in free hemoglobin concentration. The red cell lysis may be induced by complement activation. However, a free hemoglobin concentration of 53.7 ± 5.2 μmol/L found in the isoagglutinin-depleted plasma is far below the concentration of 125 μmol/L, which showed an effect of free hemoglobin on nitric oxide (NO) bioavailability in an in vivo rat model [20]. Free hemoglobin in transfused blood products like RCC or plasma will be bound on the recipients' haptoglobin [26]. After binding to haptoglobin, the hemoglobin-haptoglobin protein complex is scavenged by CD163+ macrophages and monocytes in order to clear the organism of toxic cell-free hemoglobin [27]. During storage of RCC, free hemoglobin increases as a result of hemolysis due to aging. However, a hemolysis rate of up to 0.8%, which corresponds to 220 μmol/L of free hemoglobin, greatly exceeds the levels of free hemoglobin found in the isoagglutinin-depleted plasma. The transfusion of RCC is considered to be safe due to dilution effects in patients' circulation and the binding capacity of haptoglobin. Even in massive transfusion settings, while haptoglobin levels decreased after transfusion of 1,000 mL of transfused blood, detectable levels of free hemoglobin were only found after transfusion of 2,000 mL (2 μmol/L, corresponding to 3.2 mg/dL). Even after the transfusion of 5,000 mL stored whole blood free hemoglobin levels only increased to 4 μmol/L (6.4 mg/dL) [28]. Hemolysis is defined as a free hemoglobin concentration above 30 mg/dL, while levels above 100 mg/dL are defined as severe hemolysis [29].

Although we removed the anti-B isoagglutinins from group A plasma, A-antigens are still present on several plasma proteins. Those proteins are not removed using the method proposed in this work. The presence of A-antigens on soluble proteins may lead to the formation of circulating immune complexes if the plasma is transfused to patients with anti-A. Patients who received compatible but non-identical plasma showed increased mortality compared to recipients of ABO identical plasma transfusion (relative risk, 1.15; 95% confidence interval, 1.02–1.29) [30]. This also applies to group AB plasma, which has been safely used as universal plasma for decades. Decreasing mortality may be an argument to use group O plasma for isoagglutinin depletion as it does not contain solube A and B antigens. However, the amount of red cells needed to deplete the anti-A and anti-B titers, which are usually higher in group O plasma [31, 32], might impose RCC stocks of blood group AB RCCs. High titers of anti-A and anti-B are also found in group A and B plasma. These high titers can be induced by pregnancy, vaccination, or certain food components [33, 34]. Following the German hemotherapy guidelines, plasma of females with a history of pregnancy must not be used to directly treat patients unless it is tested negative for anti-human neutrophil antigen and anti-human leukocyte antigen antibodies. Untested plasma from females with pregnancy history will not be used for the presented method. In addition, pooling of three plasma units reduces the overall titer in a plasma pool containing one high titer unit ensuring the stability of isoagglutinin depletion.

It is controversially discussed that pooling blood components of several donors increases the risk of transfusion transmitted infections (TTI) or transfusion-associated adverse effects in comparison to single donations. The risk of TTI is, at least theoretically, enhanced for pooled platelet concentrates (PCs); however, no epidemiological study or clinical trial has demonstrated different risks of viral infections transmitted by pooled PCs compared to apheresis PCs [35]. To keep the risk as low as possible we used quarantine stored plasma units of donors, who tested negative in the standard infection assays for TTI at least 4 months later during a follow-up donation. Further risk reduction can be achieved by assigning the three plasma units from one pool to the same patient. At least 15 mL of plasma per kilogram bodyweight should be transfused to treat coagulopathy (equals 900 mL or 3 units if the patient weighs 60 kg).

To lower the risk of transfusing emerging pathogens, pathogen reduction methods (e.g., amotosalen/UVA, Intercept® Blood System; riboflavin/UV, Mirasol® PRT, or methylene blue/visible light, Theraflex®-MB) can be applied to the isoagglutinin-depleted plasma before refreezing. Pathogen-reduced isoagglutinin-depleted plasma can be manufactured directly after plasma donation and does not require quarantine storage. This is of particular interest for the use of convalescent plasma from recovered donors for the treatment of new emerging pathogens.

Commercially produced units of universal plasma are currently not available in Europe and North America since regulatory authorization of Uniplas® was waived in 2009 [36] and Bioplasma FDP is only produced and used in South Africa [23]. Using the method described here, blood banks are able to cost-effectively provide isoagglutinin-depleted plasma. When summarizing the additional costs for the RCC (proportionally 40 mL for the treatment of three plasma units), tubes, bags, and tube welding for connecting the system, the price of one unit of isoagglutinin-depleted plasma increases by approximately 20% compared to fresh frozen plasma.

## Conclusion

We have described a method for the production of anti-A- and anti-B-depleted plasma in a closed system that uses standard blood processing equipment. The method was shown to be scalable using group A plasma and group B red cells. The resulting anti-A- and/or anti-B-depleted plasma may allow for blood group-independent plasma transfusion.

## Statement of Ethics

Our research complies with the guidelines for human studies and the research was conducted ethically in accordance with the World Medical Association Declaration of Helsinki. The institutional ethics review board of Universitätsmedizin Greifswald approved the study (BB 014/14). As we used plasma units found not eligible for treating patients or use for fractionation, a separate written consent was not collected.

## Conflict of Interest Statement

The Universitätsmedizin Greifswald has filed a patent for the described method of universal plasma production. A.G. is an advisor for Maco Pharma. A.G. and K.A. received funding from Maco Pharma for another project. J.R. declares no conflict of interest. M.J. declares no conflict of interest. In the study exclusively anonymized data were used. The institutional ethics review board of the University Medicine Greifswald approved the study.

## Funding Sources

This work was funded by the research budget of Universitätsmedizin Greifswald and the Deutsche Forschungsgemeinschaft (DFG [German Research Foundation] grant No. 374031971-TRR 240).

## Author Contributions

J.R. performed the experiments and analyzed the data. A.G. developed the concept and designed the study. M.J. performed the experiments and analyzed the data. K.A. designed the concept, performed the experiments, and analyzed the data. All authors contributed to writing the manuscript. All authors had full access to all data, including all statistical reports and tables used in the manuscript.

## Data Availability Statement

All data generated or analyzed during this study are included in this article. Further enquiries can be directed to the corresponding author.

## Figures and Tables

**Table 1:** Quality control parameters of the produced anti-B-depleted group A plasma

Pool No.	Plasma unit No.	Weight of single plasma units, g	Residual red cell count, ×10^9^/L	Free hemoglobin, µmol/L	Factor V^a^, %	Factor VIII^a^, %	Factor XI^a^, %	Microbiological control	Anti-B titer	
									IgM	IgG
1	1 2 3	302 282 290	0	55.8	84	93.7	81	Sterile	<1:1	<1:1

2	4 5 6	298 306 301	0	47.2	84	119.8	88	Sterile	<1:1	<1:1

3	7 8 9	296 299 289	0	52.2	99	112.2	92	Sterile	<1:1	<1:1

4	10 11 12	320 337 234	0	59.5	85	102	75	Sterile	1:1	1:1

a Plasma units were frozen and thawed once before pooling and then frozen and thawed again before testing to reflect the coagulation factor activities when anti-A-/anti-B-depleted plasma is produced from quarantine stored plasma units and then stored at −30°C until use.

**Table 2:** Quality parameters of plasma units before and after the isoagglutinin depletion treatment

	Group A plasma units before isoagglutinin depletion treatment (after freezing and thawing, *n* = 12, mean ± SD)	Group A plasma units after final isoagglutinin depletion treatment (double freezing and thawing, *n* = 12, mean ± SD)	Statistical difference, *p*
Residual red cell count, ×10^9^/L	0.5±0.08	0	>0.05
Cell-free hemoglobin, µmol/L	26.0±15.0	53.7±4.7	<0.001
Clotting factor V, %	97.8±7.3	96.3±7.8	>0.05
Clotting factor VIII, %	107.3±15.3	102.8±16.6	>0.05
Clotting factor XI, %	95.7±20.1	94.8±19.6	>0.05
